# Urinary and anal incontinence among female gymnasts and cheerleaders—bother and associated factors. A cross-sectional study

**DOI:** 10.1007/s00192-021-04696-z

**Published:** 2021-02-13

**Authors:** Kristina Lindquist Skaug, Marie Ellström Engh, Helena Frawley, Kari Bø

**Affiliations:** 1 Department of Sports Medicine, Norwegian School of Sport Sciences, PO Box 4014, Ulleval Stadion, 0806 Oslo, Norway; 2 Department of Obstetrics and Gynaecology, Akershus University Hospital, Lørenskog, Norway; 3 Faculty of Medicine, University of Oslo, Oslo, Norway; 4 Melbourne School of Health Sciences, The University of Melbourne, Melbourne, Australia

**Keywords:** Anal incontinence, Athletes, Epidemiology, Females, Stress urinary incontinence, Urinary incontinence

## Abstract

**Introduction and hypothesis:**

Artistic gymnastics, team gymnastics and cheerleading are sports including high-impact activities. It is presumed that the athletes’ pelvic floor must be functioning well to prevent urinary (UI) and anal incontinence (AI) during sports. The aim of this study was to investigate the prevalence and risk factors for UI and AI in female artistic gymnasts, team gymnasts and cheerleaders; the influence of UI and AI on daily living and sport performance; and the athletes’ knowledge about the pelvic floor muscles (PFM).

**Methods:**

All female athletes ≥ 12 years of age competing in ≥ 1 National Championship in artistic gymnastics, team gymnastics or cheerleading in 2018/2019 were invited. International Consensus on Incontinence Questionnaires were used to assess the prevalence/bother of UI and AI.

**Results:**

Among the 319 gymnasts and cheerleaders who participated, the prevalence of UI and AI was 67% and 84%, respectively. Age, training ≥ 4 days/week and straining to void were significantly associated with stress urinary incontinence (SUI) and years of training with AI. Eighty-three percent of athletes with SUI reported a negative effect on sports performance, 22% would occasionally avoid training or specific exercises because of leakage, and 28% used pads for protection. Forty-one percent of the athletes had never heard about the PFM, and 74% reported an interest in PFM training to prevent/treat UI or AI.

**Conclusions:**

UI and AI were prevalent in female gymnasts and cheerleaders, and SUI negatively influenced sport performance. The athletes’ knowledge about the PFM was limited.

## Introduction

Stress urinary incontinence (SUI) is the most common form of urinary incontinence, defined as “the complaint of involuntary loss of urine on effort or physical exertion (e.g., sporting activities), or on sneezing or coughing” [1, 2]. This definition highlights that SUI may be a condition of concern in exercising women, and high prevalence has been reported among female athletes from different sports [3]. Anal incontinence (AI) includes involuntary loss of liquid or solid stool or gas [2]. AI among female athletes is less studied than UI [3].

The main functions of the pelvic floor are to provide support to the pelvic organs (the bladder, urethra, vagina, uterus and rectum) and to counteract to increases in intra-abdominal pressure (IAP) and ground reaction forces during daily activities [4]. Artistic gymnastics, team gymnastics and cheerleading are sports including significant levels of high-impact acrobatic and gymnastic elements. Landing from 90 cm height may incur ground reaction forces up to 56.0 N/kg [5]. It is therefore presumed that gymnasts and cheerleaders need well-functioning pelvic floor connective tissue and muscles to prevent incontinence during sports. Hence, their pelvic floor may serve as a model to understand the mechanisms of pelvic floor dysfunctions (PFDs). Elite gymnasts and cheerleaders reach their top level at a young age, often as adolescents, and the clothing used in training and competition is often tight and minimal. Therefore, it is reasonable that these athletes may be especially exposed and bothered by incontinence. To date, there is scant knowledge on UI or AI in female gymnasts or cheerleaders, and there is limited knowledge on risk factors, bother and whether these conditions affect daily life and sport performance [3]. Durnea et al. [6] found that symptoms of UI at a young age (before and during pregnancy) have been shown to increase the risk of later development of and more severe UI. Early detection of incontinence in young athletes may therefore prevent further development of the condition.

The aim of this study was to investigate the prevalence of and risk factors for UI and AI in high-performance female artistic gymnasts, team gymnasts and cheerleaders and to investigate the bother of UI and AI, influence of SUI on sport performance and the athletes’ knowledge of the pelvic floor muscles (PFM).

## Methods

### Experimental approach

This was a cross-sectional study targeting all female artistic gymnasts, team gymnasts and cheerleaders at the top national junior and senior level in Norway. The study was approved by the Regional Ethics Committee (2018/2211/REK Sør-øst B, 20.12.2018) and the Norwegian Centre for Research Data (NSD: 199381, 24.01.2019). All participants or parents of athletes < 16 years old gave written informed consent.

### Subjects

Inclusion criteria were being ≥ 12 years of age and participation in ≥ 1 Norwegian National Championship (NCC) in artistic gymnastics, team gymastics or cheerleading during 2018/2019. Athletes who did not meet the inclusion criteria, reported neurological disease or previous surgery for UI or completed < 90% of the questionnaire were excluded. The response percentage was calculated by the survey software and included informative text and questions (90% response: 85/92 questions with 7 questions on knowledge of the PFM excluded). Participant lists of NCCs in 2018/2019 were used to identify eligible athletes.

### Questionnaire design, data collection and recruitment

The questionnaire was author-designed and included validated questions on incontinence. Prior to distribution to the full cohort, contact persons from the Norwegian Gymnastics Federation and the Norwegian Federation of American Sports were asked to distribute the questionnaire to a group of athletes/coaches for revision and feedback on the content. Minor revisions to questions regarding sport activities were made based on the feedback. Data were collected by an electronic questionnaire (Survey Xact) between March 2019 and June 2020. Collaboration with the two sport federations was established for recruitment of participants. Participants were recruited at four different NCCs, by email correspondence with clubs/coaches and via a registration link on the federations’ webpages and social media platforms, such as Facebook and Instagram.

### Outcome measures

Our primary outcomes were prevalence of UI and AI. Definitions of UI and AI were based on the International Urogynecological Association (IUGA)/International Continence Society (ICS) joint report on the terminology for female PFD [2]. Patient-reported outcome measures with Grade A recommendation from the International Consensus on Incontinence (ICI) 2017 were used to assess prevalence of UI and AI: the ICI Questionnaire-Urinary Incontinence Short Form (ICIQ-UI-SF) for UI and questions from the ICI Questionnaire Anal Incontinence Symptoms and Quality of Life Module (ICIQ-B) for AI [7]. Athletes were considered continent if they answered “never” to the question “When does urine leak?” and “always” to questions regarding control of watery/loose stool, formed/solid stool and wind (flatus) for UI and AI, respectively. UI was further classified into different subgroups based on participant response to the fourth question of the ICIQ-UI-SF: “When does urine leak?”. AI was classified into three subgroups: involuntary loss of gas, solid stool and liquid stool. Age, body mass index (BMI), training frequency (days/week and hours/session), level of competition (national/international), years specializing in gymnastics/cheerleading, straining at toilet, urinary tract infections and risk of female athlete triad were considered possible risk factors for UI and AI. Parity was not assessed, since we expected that participants would be of young age and nulliparous. The female athlete triad refers to the interrelationship of menstrual dysfunction, low energy availability (with or without an eating disorder) and impaired bone health and was assessed with The Low Energy Availability in Females Questionnaire (LEAF-Q) [8]. The questionnaire has demonstrated acceptable validity in classifying current energy availability, bone health and/or reproductive function in female athletes [8]. Female athletes of ≥ 15 years of age with scores ≥ 8 were considered at risk of the triad. Straining at toilet was assessed by the questions: “Do you need strain to empty your bladder?” [9] and “Do you need to strain to open your bowels?” [7]. Response alternatives from “never” to “always”/“daily” were given. To control for possible confounding of chronic disease and previous pelvic/lumbar surgery, the participants were asked to answer yes/no to the questions: “Do you have a chronic disease (e.g., diabetes, Crohn’s disease) or other health problems?” and “Have you previously had surgery in the pelvic or lumbar area?” Athletes responding “yes” were asked to add their disease or type of surgery as free text.

Bothers of UI and AI were assessed with questions from ICI questionnaires on how UI and AI affected their daily life (scale from 0 to 10). Furthermore, questions regarding UI during gymnastic and acrobatic activities, impact of UI on sports performance, and protective or preventive measures for UI during training or competition were included with provided options for responses. Some of these questions were based on a previous survey in rhythmic gymnasts [10] and others were constructed by the authors in collaboration with the sports federations. Questions from the ICIQ-B regarding sudden AI and worries of AI were added.

The questionnaire finally included questions regarding the athletes’ knowledge about the PFM. We asked if they had previously heard about the PFM and from where, if they knew how or why to train the PFM, to rate their knowledge of the PFM on a scale of 1–10 and if they were willing to do PFM training if they knew how. These questions were selected from two studies by Neels et al. [11] and Gram and Bo [10].

### Statistical analysis

Statistical analyses were performed in SPSS statistical software package version 24 (SPSS Inc., Chicago IL, USA). Background variables are presented as numbers with percentages or means with standard deviation (SD). Prevalence is reported as frequency and percentage. Pearson chi-square test was used to investigate differences in proportions of SUI/AI between the different sport groups. Risk factors for UI and AI were estimated by multivariate binary logistic regression analysis and reported as odds ratio (OR) with 95% confidence intervals (CI). The *p* value was set to 0.05. The “purposeful selection” approach was used to select variables in the multivariate logistic regression models [12]. Variables with *p* < 0.1 were left in the final model. Categorical variables with more than two categories were recoded into dichotomous variables (straining on voiding, training frequency/week, urinary tract infection). Choice of reference group when comparing risk between the different sport groups was based on results from the chi-square test. Continuous variables showing non-linear associations with SUI/AI in univariate analysis were recoded into ordinal variables based on quartiles. No power calculations were made, since we aimed to include all athletes fulfilling the inclusion criteria.

## Results

**Fig. 1:**
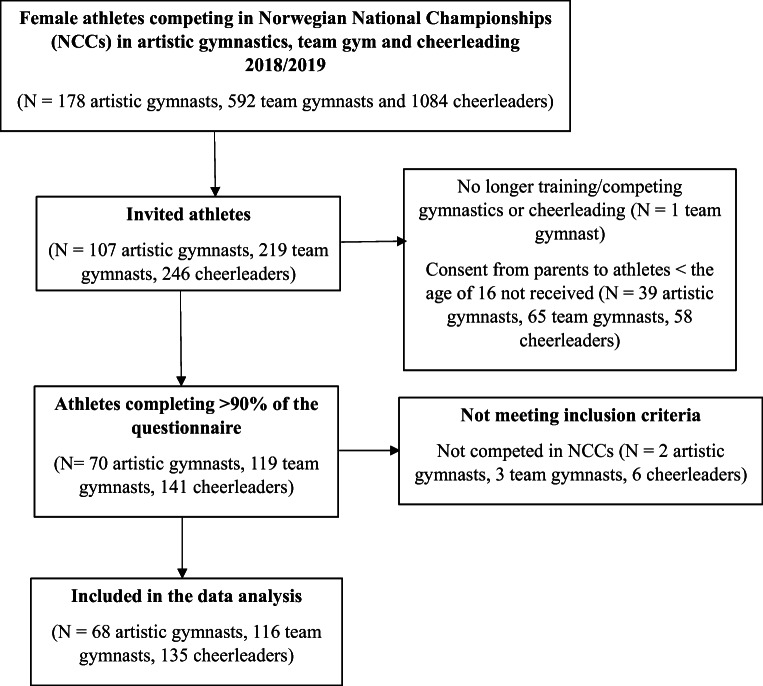
Flow chart of participant enrollment

One hundred seventy-eight female artistic gymnasts, 592 team gymnasts and 1084 cheerleaders were identified from participation lists of NCCs during 2018/2019. Of these, we were able to invite 107 artistic gymnasts (60.1%), 219 team gymnasts (37.0%) and 246 cheerleaders (22.7%) to the study. Finally, 68 artistic gymnasts, 116 team gymnasts and 135 cheerleaders were included, resulting in a response rate of 38.2%, 19.6% and 12.5%, respectively. Four hundred twenty-seven (99.5%) athletes completed 100% and two > 90% of the questionnaire. The number of participants at each stage of the inclusion process is presented in Fig. 1.

Background data, medical and sport practice characteristics are presented in Table 1 and prevalence of UI, AI and subtypes of UI/AI in Table 2. High prevalence of both UI and AI were reported among all sports. There were no differences in proportions of SUI between the artistic gymnasts and team gymnasts (*p* = 0.14). The proportion of SUI was significantly lower in cheerleaders compared to artistic gymnasts (*p* < 0.001) and team gymnasts (*p* < 0.001). No differences were found when comparing proportions of AI in artistic gymnasts and team gymnasts (*p* = 0.48), artistic gymnasts and cheerleaders (*p* = 0.91) or team gymnasts and cheerleaders (*p* = 0.48).

**Table 1:** Sociodemographic, anthropometric, medical and sport practice characteristics of female artistic gymnasts, team gymnasts and cheerleaders

	Total, *n* = 319	Artistic gymnasts, *n* = 68	Team gymnasts, *n* = 116	Cheerleaders, *n* = 135
Age (years), mean (SD, min–max)	17.4 (3.2, 12–36)	16.8 (3.6, 12–36)	17.1 (2.7, 13–28)	17.9 (3.3, 12–29)
BMI^1^, mean (SD, min–max)	21.7 (2.7, 14.6–37.2)	21.1 (2.5, 16.0–28.2)	21.7 (2.2, 16.0–31.0)	21.9 (3.1, 14.6–37.2)
Gymnastic/cheerleading training				
Days/week, N (%)				
1–3 days	70 (21.9)	5 (7.4)	8 (6.9)	57 (42.2)
4–5 days	203 (63.6)	26 (38.2)	102 (87.9)	75 (55.6)
6–7 days	46 (14.4)	37 (54.4)	6 (5.2)	3 (2.2)
Hours/session, mean (SD, min–max)	2.6 (0.6, 1–6)	3.3 (0.7, 2–5)	2.6 (0.4, 2–6)	2.2 (0.4, 1–3.25)
Years specializing in gymnastics/cheerleading, mean (SD, min–max)	6.8 (3.5, 0–28)	8.6 (4.3, 2–28)	6.6 (3.3, 1–17)	6.1 (2.7, 0–14)
Level of competition, N (%)				
National	176 (55.2)	54 (79.4)	72 (62.1)	50 (37.0)
International	143 (44.8)	14 (20.6)	44 (37.9)	85 (63.0)
Menarche, N (%)	294 (92.2)	58 (85.3)	110 (94.8)	126 (93.3)
Risk of female athlete triad, N (%)	122 (38.2)	22 (32.4)	54 (46.6)	46 (34.1)
Chronic disease, N (%)	47 (14.7)	7 (10.3)	12 (10.3)	28 (20.7)
Previous surgery in pelvic/lower back area, N (%)	7 (2.2)	0 (0)	1 (0.9)	6 (4.4)
Urinary tract infections, N (%)				
Never	291 (91.2)	64 (94.1)	113 (97.4)	114 (84.4)
1–3/year	23 (7.2)	4 (5.9)	2 (1.7)	17 (12.6)
4–12/year	5 (1.6)	0 (0)	1 (0.9)	4 (3.0)
> 1/month	0 (0)	0 (0)	0 (0)	0 (0)
Straining to void, N (%)				
Never	115 (36.1)	31 (45.6)	37 (31.9)	47 (34.8)
Occasionally	145 (45.5)	26 (38.2)	56 (48.3)	63 (46.7)
Frequently	45 (14.1)	9 (13.2)	18 (15.5)	18 (13.3)
Daily	14 (4.4)	2 (2.9)	5 (4.3)	7 (5.2)
Straining to defecate, N (%)				
Never	10 (3.1)	4 (5.9)	0 (0)	6 (4.4)
Rarely	110 (34.5)	30 (44.1)	39 (33.6)	41 (30.4)
Some of the time	167 (52.4)	30 (44.1)	64 (55.2)	73 (54.1)
Most of the time	31 (9.7)	4 (5.9)	12 (10.3)	15 (11.1)
Always	1 (0.3)	0 (0)	1 (0.9)	0 (0)

^1^Total N for BMI was 315 due to missing data

**Table 2:** Prevalence of urinary and anal incontinence in female artistic gymnasts, team gymnasts and cheerleaders

	Total, *n* = 319 N (%)^1^	Artistic gymnasts, *n* = 68 N (%)^2^	Team gymnasts, *n* = 116 N (%)^3^	Cheerleaders, *n* = 135 N (%)^4^
Overall UI	215 (67.4)	48 (70.6)	97 (83.6)	70 (51.9)
SUI	201 (63.0)	48 (70.6)	93 (80.2)	60 (44.4)
UUI	31 (11.6)	6 (8.8)	15 (12.9)	16 (11.9)
MUI	30 (9.4)	6 (8.8)	13 (11.2)	11 (8.1)
Other UI	32 (10.0)	6 (8.8)	13 (11.2)	13 (9.6)
Overall AI	268 (84.0)	56 (82.4)	100 (86.2)	112 (83.0)
Liquid	130 (40.8)	25 (36.8)	48 (41.4)	57 (42.2)
Solid	39 (12.2)	8 (11.8)	16 (13.8)	15 (11.1)
Gas	259 (81.2)	56 (82.4)	97 (83.6)	106 (78.5)

AI, anal incontinence; MUI, mixed; SUI, stress UI; UUI, urgency UI; UI, urinary incontinence

^1^Percentage of total N, ^2^percentage of total N of artistic gymnasts, ^3^percentage of total N of team gymnasts, ^4^percentage of total N of cheerleaders

Results from multivariate logistic regression analysis of possible risk factors and SUI/AI are presented in Table 3. Cheerleading was chosen as the reference group when comparing the different sport groups and risk of SUI. A non-linear relationship between age and SUI was found in the univariate analysis, and a recoded ordinal variable for age (based on quartiles) was used in the multivariate regression model. Gymnastic/cheerleading training ≥ 4 days per week and straining to void were found to be significantly associated with SUI. Athletes aged 16 or 17 years had significantly higher odds of SUI than younger athletes (12–15 years), and cheerleaders had significantly lower odds compared to both artistic gymnasts and team gymnasts. No significant differences in odds of SUI were found when comparing team gymnasts with artistic gymnasts (OR: 1.82, 95% CI: 0.87–3.80, *p* = 0.11). Years with specialization in gymnastics/cheerleading was the only variable found to be positively associated with AI.

**Table 3:** Odds ratios with 95% confidence intervals of risk factors for stress urinary incontinence and anal incontinence in female artistic gymnasts, team gymnasts and cheerleaders (*n* = 319)

	B	OR (95% CI)	*p* value
SUI			
Age			
12–15 years^1^			
16 years (1)	1.24	3.45 (1.66–7.18)	0.001
17 years (2)	1.64	5.18 (2.15–12.48)	<0.001
≥ 18 years (3)	0.48	1.62 (0.83–3.17)	0.157
Gymnastic/cheerleading training ≥ 4 days/week			
No		1.00 (−)	
Yes	0.84	2.31 (1.22–4.37)	0.010
Straining to void	0.98	2.66 (1.26–5.64)	0.011
Type of sport			
Cheerleading^1^		1.00 (−)	
Team gymnastics (1)	1.40	4.07 (2.15–7.69)	< 0.001
Artistic gymnastics (2)	1.01	2.75 (1.39–5.46)	0.004
AI			
Years specializing in gymnastics/cheerleading	0.13	1.14 (1.02–1.26)	0.016
Chronic disease			
No		1.00 (−)	
Yes	1.10	3.00 (0.88–10.15)	0.078
Straining to defecate			
No		1.00 (−)	
Yes	0.14	3.06 (0.70–13.46)	0.137

^1^Reference group

AI: anal incontinence, B: regression coefficient, CI: confidence interval, OR: odds ratio, SUI: stress urinary incontinence

**Fig. 2:**
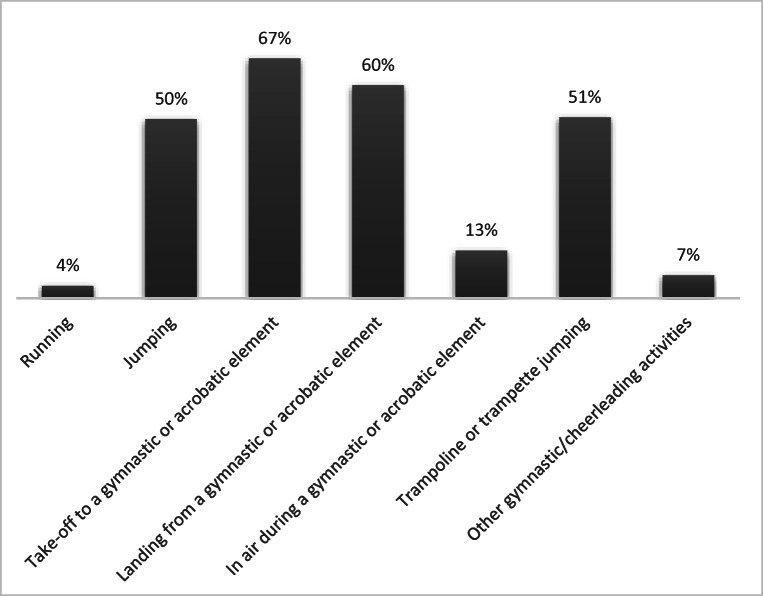
Gymnastic/cheerleading activities provoking urinary leakage among female artistic gymnasts, team gymnasts and cheerleaders with stress urinary incontinence (*n* = 201)

Among athletes reporting any UI, 107 (49.9%) experienced leakage once a week or less often, 65 (30.2%) two or three times per week, 14 (6.5%) once a day, 16 (7.4%) several times a day and 3 (1.4%) all the time. Ten (4.7%) had not experienced any leakage during the past 4 weeks. The amount of leakage was reported as small by 175 (81.4%) and moderate by 23 (10.7%). Mean ICIQ-UI-SF score was 6.3 (SD: 3.7, range: 0–17), and mean impact of UI on daily activities was 2.5 (SD: 2.4, range: 0–10), with 46 (21.4%) scoring ≥ 5. Most (*n* = 199, 99.0%) athletes with SUI experienced leakage during gymnastics or cheerleading, with take-off and landing from gymnastic/acrobatic elements reported as the most provocative activities (Fig. 2).

**Fig. 3:**
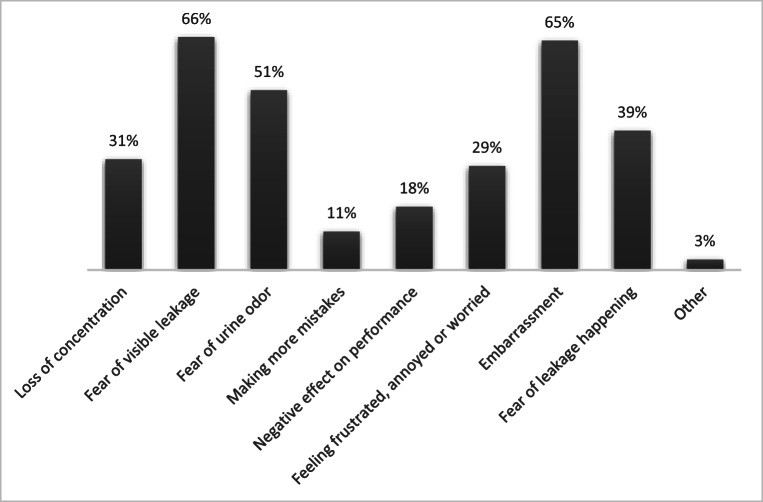
Impact of urinary leakage on sport performance in female artistic gymnasts, team gymnasts and cheerleaders with stress urinary incontinence (*n* = 201)

One hundred sixty-six (82.6%) of those with SUI reported a negative effect of UI on sports performance. Fear of visible leakage and embarrassment were the most common complaints (Fig. 3).

Most athletes with SUI reported leakage during training (*n* = 198, 98.0%) followed by during competition (*n* = 90, 44.8%). Fifty-seven (28.4%) reported use of pads to protect against visible leakage. Reported measures to prevent leakage were voiding before training/competition (*N* = 134, 66.7%), decreased fluid intake (*N* = 17, 8.5%), use of an intra-vaginal tampon (*N* = 9, 4.5%) and other measures (*N* = 7, 3.5%), such as multiple toilet visits during training or PFM training. Forty-five (22.4%) reported they would occasionally avoid training or specific exercises because of leakage. Fifty-three (26.4%) had never spoken about the condition with anyone, 13 (6.5%) had spoken with their coach and 12 (6.0%) with health care personnel, such as a physician or physiotherapist. One hundred fifteen (57.2%) had spoken about urinary leakage with their teammates, 76 (37.8%) with friends and 40 (19.9%) with a parent.

Of females reporting AI, mean bother of accidental loss of gas, liquid and solid stool was 3.0 (SD: 2.6, range: 0–10), 2.3 (SD: 2.3, 0–10) and 2.4 (SD: 2.4, range 0–10), respectively. The number of athletes scoring ≥ 5 on bother was 69 (26.6%) for loss of gas, 20 (15.4%) for loss of liquid stool and 6 (15.4%) for solid stool. Fifty-six (20.9%) reported that bowel leakage could happen occasionally or more often without warning. and 13 (4.9%) reported that they sometimes or more often were worried about bowel leakage.

Among athletes reporting accidental loss of gas, 227 (87.6%) experienced leakage during training and/or competition: 99 (38.2%) rarely, 91 (35.1%) occasionally, 31 (12.0%) often and 6 (2.3%) all the time. Of those reporting liquid AI, 24 (18.5%) rarely and 5 (3.8%) occasionally experienced leakage during training and/or competition. Of those with solid AI, 7 (17.9%) reported leakage during training/competition and all experienced it rarely.

One hundred thirty-two (41.4%) of the athletes had never heard about the PFM. Thirty-nine (12.2%) of the athletes reported that they had heard about the PFM from their coach, 32 (10.0%) from teammates, 61 (19.1%) from health personnel and 54 (16.9%) from other sources (friends, siblings or parents). The mean self-rated knowledge of the PFM was 1.5 (SD: 1.7) of 10. Thirty-two (10.0%) knew how and 58 (18.2%) why to train the PFM. Two hundred thirty-five (73.7%) responded they would do PFM training to prevent or treat UI and AI if they knew how. Three athletes (0.9%) reported they did or had tried PFM training. Responses on knowledge of the PFM were lacking from two athletes.

## Discussion

The aim of this study was to investigate the prevalence and risk factors for UI and AI in high-performance gymnasts and cheerleaders. Furthermore, we aimed to investigate bother of UI and AI, influence of SUI on sport performance and the athletes’ knowledge of the PFM. As far as we have ascertained, this is the first study including questions on the prevalence, risk factors and bother of both UI and AI, in addition to the influence on sport performance, in these sports.

We found a high prevalence of both UI (67.4%) and AI (84.0%) among female gymnasts and cheerleaders. Self-reported bothers of UI and AI were low, but most athletes with SUI reported that leakage negatively influenced sport performance. Training frequency (≥ 4 days per week), age of 16 or 17 years, straining to void and type of sport (artistic gymnastics and team gymnastics) were significantly associated with SUI and years with gymnastic or cheerleading participation with AI. Overall, the athletes’ knowledge of the PFM was low.

We did not include a control group of non-athletes in our study. However, much lower prevalence rates of UI (12–13%) have been reported in large studies of nulliparous young women [13, 14], and female athletes/exercisers have been found to have a three times increased risk of UI compared to non-exercisers [3]. In previous studies of UI in artistic gymnasts, slightly lower prevalence rates (56 and 67%) have been reported [15, 16]. The extremely high prevalence rates of UI found in our study, especially in gymnasts, is comparable to those (73 and 80%) reported among young female trampolinists [17, 18]. A similarity between these sports is the significant level of high-impact acrobatic activity, including elements with somersaults, twist and turns. In cheerleaders, we also found higher prevalence of both UI and AI than reported in a recent study (UI: 27%, AI: 63%) [19]. As in our study, SUI and gas incontinence was the most common subtype. In their study, the level of competition was not reported, and the athletes had less cheerleading experience compared to the high national level cheerleaders in our study. Hence, the studies may not be directly comparable.

Our results showed that athletes who trained ≥ 4 times per week had 2.3 times higher odds of SUI than those who trained less. Higher training dosage has also been associated with UI in studies of female trampolinists [17, 18] and athletes from different sport modalities [20], indicating that higher training exposure may increase the risk of UI. As in our study, one of the above-mentioned studies [18] found a higher prevalence of UI with increasing age. Gymnasts and cheerleaders of 16 and 17 years of age may have a higher training dosage, but also increased weight and hormonal changes due to puberty may explain the associations between age and SUI. Further studies are warranted to explore such associations in adolescent athletes. The higher odds of SUI found in artistic and team gymnasts compared to cheerleaders could possibly be explained by differences in training and competition surfaces, characteristics of the acrobatic/gymnastics elements and the forces applied on the athletes in the different sport types. However, this needs further investigations. We also found that athletes who strained to void had significantly increased odds of SUI. Straining to void has also been associated with UI among female ex-trampolinists (OR: 1.8, 95% CI: 1.1–3.4, *p* = 0.03) [21]. Anecdotally, straining to void may be a bad habit caused by women not giving themselves time to relax and trying to increase their flow rate when voiding. The flow rate is increased by abdominal straining, Valsalva or suprapubic pressure [2] and may further explain the association with SUI. A possible explanation of why these athletes strain to void may be a hypertonic or non-relaxing pelvic floor. This condition may lead to impaired ability to evacuate urine or stool and has been suggested by Louis-Charles et al. [22] to be a condition affecting female athletes. However, research on hypertonicty or inability to relax the PFM during voiding is limited both in the general population and in athletes, and we have no data in our study on the athletes’ PFM. We found no associations between SUI and other variables (BMI, level of competition, years specializing in gymnastics/cheerleading, urinary tract infections, risk of female athlete triad). In other studies of female athletes, associations among BMI, eating disorders/female athlete triad, training dosage and UI have been studied, but the results are inconsistent [3].

The only factor found to be associated with AI in our study was the number of years specializing in gymnastics/cheerleading. To date, there are few other studies of AI in female athletes [3]. The high prevalence rates reported in our study demonstrate a need for further studies of possible risk factors and mechanisms of AI in high-impact athletes.

High, repetitive increases in intraabdominal pressure (IAP) have been proposed as a mechanism leading to increased risk of UI in athletes participating in high-impact sports [3]. No studies of IAP and gymnastics or cheerleading were found. However, in a study by Seegmiller et al. [5], ground reaction forces produced during drop landings were found to be higher in high-level gymnasts compared to recreational athletes. An opposing hypothesis is that impact during exercise can lead to co-contractions of the PFM and create a strengthening effect on the pelvic floor and further reduced risk of PFD [3]. However, in a recent randomized controlled trial (RCT) by Luginbuehl et al. [23], “involuntary reflexive PFM training” (e.g., jumping exercises) did not produce any additional treatment effects in reducing UI symptoms compared to standard strength training of the PFM. In our study, the prevalence of UI and AI were high despite the great amount of high-impact training. In addition, jumping and landing from acrobatic or gymnastic elements were reported as the activities provoking the most urinary leakage. These results indicate that high-impact exercise training cannot prevent or treat PFD.

Fatigue of the PFM during exercise could be another possible mechanism of SUI in gymnasts and cheerleaders. In a cross-over study of young women with SUI, Ree et al. [24] found that 90 min of heavy exercise (lifting and jumping/running) reduced maximum voluntary PFM contractions by 17%, and Middlekauff et al. [25] found that high-intensity CrossFit exercises caused an immediate descent of the pelvic floor. To our knowledge, no studies have evaluated the immediate or long-term effect of gymnastic and cheerleading activity on the pelvic floor. Given the high impact on the pelvic floor in these athletes, it is presumed that they require much better function of the PFM and connective tissue than non-exercisers to prevent UI and AI. The fact that UI and AI did not seem to bother them during daily activities indicates that the impact during such activities was not enough to induce incontinence and leakage may be mostly related to sport activities.

Most of the athletes with SUI in our study reported negative effects on performance; > 60% reported that leakage led to embarrassment. This could explain why few had discussed their condition with coaches or medical personnel. Fear of visible leakage was another common concern. This was also reported as the most common complaint by rhythmic gymnasts with SUI [10]. In these sports, athletes wear tight and minimal clothes; therefore, signs of leakage may be especially visible. In other studies, female athletes have reported a negative effect of UI on the performance and quality of life [26, 27], and for some athletes, UI has led to avoidance or cessation of sport activity or exercise [21, 27]. The latter was also found in the present study where about 1/5 of the athletes with SUI reported they would occasionally avoid training or specific exercises due to leakage.

The gymnasts and cheerleaders in our study had limited knowledge about the PFM. Although few of those with SUI had spoken with their coach or medical personnel about the condition, most of the athletes reported an interest in PFM training to prevent or improve incontinence. This is in line with findings from a study of female college athletes [28], indicating that few athletes seek advice on how to treat or prevent UI or other PFD.

Strength training of the PFM has been shown to be effective in treating UI in women and is recommended by international clinical practice guidelines as first-line treatment (evidence level 1, recommendation Grade A) [29]. However, evidence of the effect of PFM training in female elite athletes is limited. In a RCT of 32 female volleyball players, PFM training showed significant improvement of UI compared to written information only [30]. However, based on current knowledge we do not know whether PFM training is effective in athletes exposed to excessive impact during gymnastics or acrobatics. Possible effects should be investigated in future high-quality RCTs.

Strengths of the present study were the inclusion of top-level athletes from different high-impact gymnastic and acrobatic sports and assessment of possible risk factors, bother and athletes’ knowledge about the pelvic floor. We used valid and reliable questionnaires to collect data on UI and AI [7], and the total number of participants compares favorably with other studies of young female athletes [10, 15–18, 20].

A limitation of our study was the low response rate, with a possible selection bias and further influence on the external validity. Our results were based on self-reported measures, and no clinical measures were used to verify PFD or possible risk factors. As for all cross-sectional studies, exposure and outcome were measured at the same time point, and a cause-effect relation cannot be inferred.

## Conclusion

Our study found that UI and AI were highly prevalent in female gymnasts and cheerleaders. Higher training frequency was found to be associated with SUI and years with gymnastic/cheerleading experience with AI, indicating an increased risk of UI/AI with higher training exposure. The mechanisms of UI/AI in gymnasts and cheerleaders are to date unknown, and studies investigating the mechanistic effect of high-impact acrobatics and gymnastics on the pelvic floor are warranted. Most athletes with SUI reported that urinary leakage negatively influenced sport performance. Research to test interventions to treat/prevent PFD in these athletes, such as PFM training, is urgently required.
